# Progress in the research of p53 tumour suppressor activity controlled by Numb in triple‐negative breast cancer

**DOI:** 10.1111/jcmm.15366

**Published:** 2020-06-05

**Authors:** Jie Xian, Yu Cheng, Xue Qin, Yijia Cao, Yetao Luo, Youde Cao

**Affiliations:** ^1^ School of Basic Medical Sciences Medical University of Chongqing Chongqing China; ^2^ School of Laboratory Medicine Medical University of Chongqing Chongqing China; ^3^ Breast Surgery Chongqing Traditional Chinese Medicine Hospital Chongqing China; ^4^ School of Public Health and Management Medical University of Chongqing Chongqing China

**Keywords:** HDM2, Numb, p53, triple‐negative breast cancer

## Abstract

Numb is known as a cell fate determinant as it identifies the direction of cell differentiation via asymmetrical partitioning during mitosis. It is considered as a tumour suppressor, and a frequent loss of Numb expression in breast cancer is noted. Numb forms a tri‐complex with p53 and E3 ubiquitin ligase HDM2 (also known as MDM2), thereby preventing the ubiquitination and degradation of p53. In this study, we examined Numb expression in 125 patients with triple‐negative breast cancer (TNBC). The results showed that 61 (48.8%) patients presented with a deficient or decreased Numb expression. The percentage of Ki67 > 14% in the retained Numb group was significantly lower than that in the decreased and deficient Numb groups (86.00% vs. 98.40%, *P* = .0171). This study aimed to detect the expression and migration of Numb, HDM2 and p53 in the membrane, cytoplasmic and nuclear fractions of normal mammary epithelial cell line MCF‐10A and basal‐like TNBC cell line MDA‐MB‐231. We obtained the cell fractions to identify changes in these three protein levels after the re‐expression of *NUMB* in the MDA‐MB‐231 cells and the knocking down of *NUMB* in the MCF‐10A cells. Results showed that Numb regulates p53 levels in the nucleus where the protein levels of Numb are positively correlated with p53 levels, regardless if it is re‐expressed in the MDA‐MB‐231 cells or knocked down in the MCF‐10A cells. Moreover, HDM2 was remarkably decreased only in the membrane fraction of NUMB knock‐down cells; however, its mRNA levels were increased significantly. Our results reveal a previously unknown molecular mechanism that Numb can migrate into the nucleus and interact with HDM2 and p53.

## 1 INTRODUCTION

Numb was first discovered in the Drosophila sensory neural precursor cells (SOPs).^1^ The asymmetrical partitioning of Numb in the division of SOPs determines the differentiation direction of daughter cells; therefore, it is called the cell fate determiner.^2^, ^3^, ^4^ The mammary epithelium comprises an inner layer of luminal epithelial cells and an outer layer of myoepithelial cells with mesenchymal characteristics.^5^ Numb and Numb‐like (NumbL) are abundantly expressed in the mammary muscle epithelial cells; their levels reach a peak in mice during pregnancy.^6^ Moreover, the knocking out of Numb and NumL in mice can damage mammary muscle epithelial cells and promote epithelial‐to‐mesenchymal transition leading to an unsuccessful lactation.^7^, ^8^ Furthermore, the loss of Numb expression was associated with the occurrence of breast cancer. Decreased Numb expression also affects cell cycle proteins, thus accelerating the transformation of G1/S phase and promoting the proliferation of tumour cells.^9^ Breast cancer is a common malignant tumour in women, and its incidence rate has been increasing annually. Triple‐negative breast cancer (TNBC) is a type of malignant breast cancer that represents a sub‐group of breast cancers that tests negative for oestrogen receptors (ER), progesterone receptors (PR) and human epidermal growth factor receptor 2 (HER2).^10^, ^11^ In breast cancers, a significant positive correlation was noted between Numb expression (deficient and decreased vs. retained) and ER and PR status.^12^ The immunohistochemical staining of the pathological sections from 241 patients with breast cancer revealed that approximately 44% of deficient or decreased Numb expression was noted in 25 patients with basal‐like TNBC, and its loss or decrease was associated with the degree of tumour malignancy, prognosis and 5‐year survival rate.^12^ We performed immunohistochemical staining on the paraffin sections from 125 Chinese patients with TNBC who were diagnosed at the Clinical pathology diagnosis centre of Medical University of Chongqing between 2012 and 2017 to detect Numb expression. The results demonstrated that 61 (48.8%) patients presented with deficient or decreased Numb expression. The percentage of Ki67 >14% in the retained Numb group was significantly lower than that in the decreased and deficient Numb groups (*P* = .0171). However, no significant difference was noted in terms of age, tumour size, lymph node status and histological type between the retained Numb and decreased and deficient Numb groups.

The mutation and decrease of the tumour suppressor p53 in breast cancer is common. HDM2 oncoprotein can degrade P53, resulting in shorter half‐life and decreased p53 activity.^13^, ^14^, ^15^, ^16^ Numb interacts with HDM2 and prevents the ubiquitination and p53 degradation, thus maintaining the activity and stability of p53. However, the specific mechanism of underlying their interactions remains unclear.^17^ The present study aimed to further explore the migration and expression of Numb, HDM2 and p53 in the cell membrane, cytoplasm and nucleus of MCF‐10A and MDA‐MB‐231 cells. Our results demonstrated that Numb and HDM2 were detected in the cell membrane, cytoplasm and nucleus and that p53 was primarily distributed in the nucleus of the MCF‐10A and MDA‐MB‐231 cells. Furthermore, Numb expression was significantly decreased in the basal‐like TNBC cell line MDA‐MB‐231. We then re‐expressed NUMB in MDA‐MB‐231 cells and knocked down NUMB in MCF‐10A cells using short interfering RNA (siRNA) to detect changes in the three protein levels in different cell fractions. The NUMB–EGFP‐transfected MDA‐MB‐231 cells showed a twofold higher level of p53 and Numb in the nucleus. Meanwhile, no significant changes were observed in the HDM2 levels. These results demonstrated that Numb can migrate into the nucleus to inhibit the degradation of p53 by HDM2 in the NUMB–EGFP‐transfected cells. In the nucleus of NUMB knock‐down cells, the expression of Numb and p53 significantly increased, and no changes were observed in the HDM2 and p53 mRNA levels. Importantly, these results validated that Numb regulates p53 levels in the nucleus and that it is positively correlated with p53 levels.

## 2 MATERIALS AND METHODS

### 2.1 Clinical samples

We collected archival formalin‐fixed paraffin‐embedded mammary tissue specimens from 125 patients with TNBC who were diagnosed at the Clinical pathology diagnosis centre of Medical University of Chongqing between 2012 and 2017. Numb status was attributed to the tumours by measuring the levels of Numb expression by immunohistochemical staining (IHC). Normal mammary tissues showed a strong and homogeneous NUMB expression. Tumours were classified based on an IHC scale from 0 to 2 (score 2, <10% positive; score 1, 10%–50% positive and score 0, >50% positive tumour cell expression of Numb).

### 2.2 Ethics statement

The study was approved by an ethics committee and follows the tenants of the Declaration of Helsinki. This study was a retrospective clinical study. All patients had signed an informed consent before surgery and agreed to use pathological specimens for scientific research.

### 2.3 Cell culture, plasmids and reagents

MCF‐10A and MDA‐MB‐231 cell lines were obtained from the American Type Culture Collection. MCF‐10A cells were cultured in MEBM (CC‐3151, Lonza, Basel, Switzerland) supplemented with MEGM^®^ SingleQouts^®^ (CC‐4136, Lonza) and 100 ng/mL of cholera toxin. MDA‐MB‐231 cells were cultured in DMEM comprising 10% FBS (Corning). The plasmid of plRES2‐EGFP‐NUMB (PPL00760‐2a) was purchased from the Public Protein/Plasmid Library. We used Lipofectamine 2000 (180 423, GenePharma, Shanghai, China) to transfect MDA‐MB‐231 cells.

### 2.4 Immunohistochemistry

Serial sections were cut from paraffin blocks, deparaffinized with xylene and then rehydrated in a graded ethanol series. For antigen retrieval, the samples were microwaved for 14 minutes in a citrate buffer (pH = 6). Subsequently, the sections were treated with 3% hydrogen peroxide for 15 minutes. All sections were incubated with Anti‐NUMB monoclonal antibody (1:100, ab14140; Abcam) overnight at 4°C. These antibodies were identified using a biotinylated secondary antibody (PV‐9001, Beijing Sequoia Jinqiao) labelled with streptavidin‐horseradish peroxidase and a DAB staining kit (KIT‐5020, Maixin Biotechnology. As for the negative control, the primary antibody was substituted with phosphate‐buffered saline (PBS). Human adrenal gland tissue is the positive control recommended by the antibody, and it is the reliable basis to verify the quality of the antibody.

### 2.5 Immunofluorescence

Cells were seeded onto glass coverslips in 24‐well plates, washed with PBS, fixed in 4% paraformaldehyde for 20 minutes at room temperature and permeabilized with 0.1% Triton X‐100. They were incubated in blocking buffer (PBS with 5% bovine serum albumin [BSA]) for 30 minutes and then with primary antibody overnight in PBS at 4°C. Thereafter, the samples with secondary antibodies (1:100, bs‐0296G‐Cy3/bs‐0295G‐FITC; Bioss, Beijing, China) were incubated for 1 hour and stained with DAPI for 10 minutes at room temperature.

### 2.6 Cell fractionation experiments and immunoblotting

The cytoplasmic, membrane and organelle and cytoskeletal and nuclear fractions of cells were obtained by lysing using the Cell Fractionation Kit (#9038, Cell Signaling Technology). Whole‐cell proteins were extracted by lysing in RIPA buffer. The protein lysate was separated using 10% sodium dodecyl sulphate‐polyacrylamide gel electrophoresis and electro‐transferred onto a polyvinylidene fluoride membrane. The membranes were blocked in 5% BSA and were incubated with primary antibodies overnight at 4°C. The following antibodies were used: mouse monoclonal anti‐NUMB (1:1000, ab14140; Abcam), mouse monoclonal anti‐MDM2 [2A10] (1:50, ab16895; Abcam, Cambridge, MA, USA), Rabbit Polyclonal anti‐P53 (1:1000, #9282; Cell Signaling Technology), Rabbit Monoclonal anti‐Histone H3 (D1H2) (1:2000, #4499; Cell Signaling Technology), Rabbit Polyclonal anti‐Caveolin‐1 (1:1000, bs‐1453R; Bioss) and mouse anti‐GAPDH (1:1000, 60 004‐1‐Ig; Proteintech Group). The membranes were washed and incubated with anti‐rabbit or anti‐mouse secondary antibody (1:5000, SSA004/SSA007; Sino Biological Inc) for 2 hours at room temperature. Finally, antigen‐antibody complexes were detected using an electrochemiluminescence Western blotting detection reagent.

### 2.7 RNA isolation, q‐PCR and siRNA

Total RNA was isolated with RNAiso Plus (TaKaRa, Kusatsu) and reverse transcribed with PrimeScript^®^ RT reagent Kit with gDNA Eraser (Perfect Real‐Time; TaKaRa, Kusatsu). Quantitative polymerase chain reaction (q‐PCR) quantification was performed using SYBR^®^ Premix Ex Taq™ II (Tli RNaseH Plus; TaKaRa, Kusatsu) on the CFX96 Real‐Time PCR Detection System (Bio‐Rad Laboratories Inc, Hercules, California, USA). The following primers were used for q‐PCR: NUMB: sense primer 5′‐GGACACAGGTGAAAGGTTGAGC‐3′ and anti‐sense primer 5′‐AGTGGCTGTTGTGACACGGAAT‐3′; MDM2: sense primer 5′‐CTACAGGGACGCCATCGAATC‐3′ and anti‐sense primer 5′‐TGAAGTGCATTTCCAATAGTCAGC‐3′; P53: sense primer 5′‐TGCGTGTTTGTGCCTGTCCT‐3′ and anti‐sense primer 5′‐AGTGCTCGCTTAGTGCTCCCT‐3′ and GAPDH: sense primer 5′‐CTTTGGTATCGTGGAAGGACTC‐3′ and anti‐sense primer 5′‐GTAGAGGCAGGGATGATGTTCT‐3′. The reaction conditions were 95°C for 30 seconds, followed by 40 cycles at 95°C for 5 seconds and 58°C for 30 seconds. The housekeeping gene GAPDH was used for normalization, and all reactions were performed in triplicates. The relative mRNA expression was analysed using the 2‐δΔCt method. For siRNA experiments, the delivery of siRNA oligos was achieved using siRNA‐mate (180 426, GenePharma, shanghai, China). The targeted sequences were as follows: Numb siRNA, GGUUAAGUACCUUGGCCAUTT; AUGGCCAAGGUACUUAACCTT (5′ to 3′).

### 2.8 Statistical analysis

Student's t test (two‐tailed) was used to determine the significance of the differences between the groups. A *P* value of <.05 was considered statistically significant. NUMB expression intensities in human breast cancer samples were analysed using chi‐square and Fisher's exact tests. The SAS version 9.4 software (Copyright^©^ 2016 SAS Institute Inc Cary) was used for analysis. A significant difference was determined at α = 0.05.

## 3 RESULTS

### 3.1 Numb expression in normal mammary tissue and triple‐negative breast cancers

**Figure 1:**
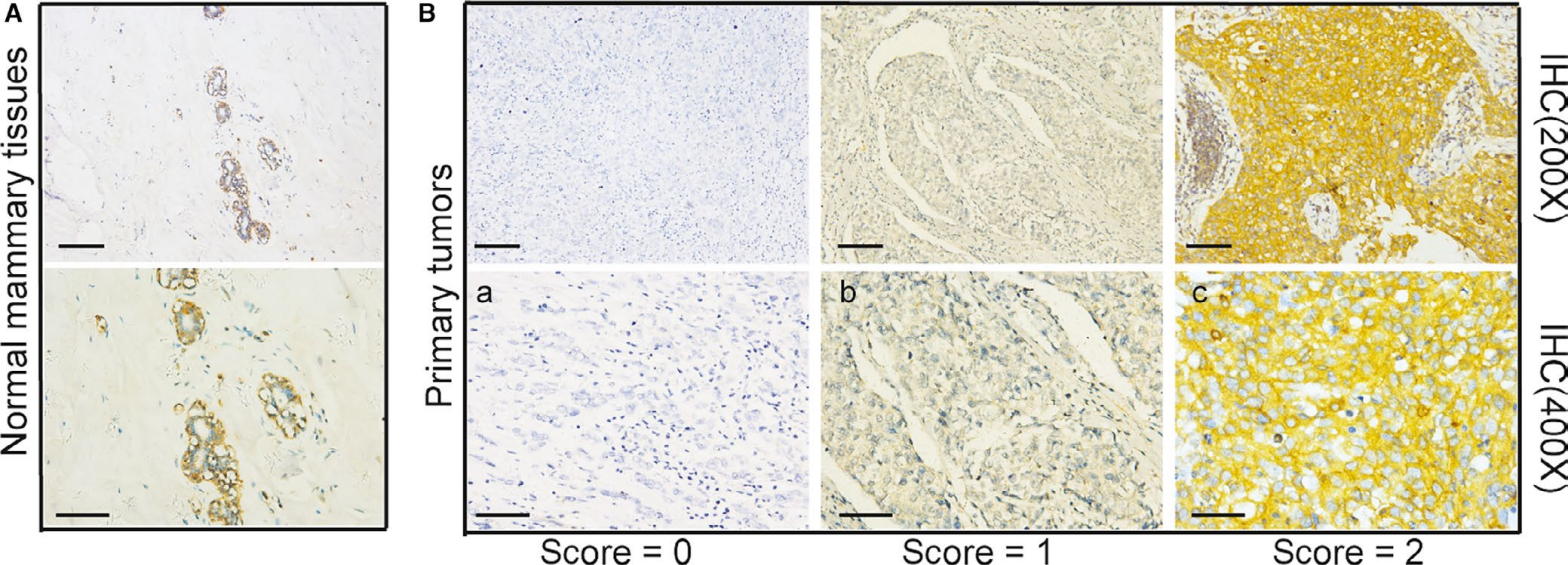
Numb expression in Normal breast tissue and Triple‐negative breast cancers. A, The expression of Numb in Normal mammary tissue detected by immunohistochemistry. B, Examples of a retained (score 2, <10% positive tumor cells), b reduced (score 1, 10%‐50% positive tumor cells) and c deficient (score 0, >50% positive tumor cells), expression of Numb

We performed immunohistochemical staining on the paraffin sections from 125 patients with TNBC to detect the Numb expression. Of the 125 patients evaluated for Numb expression, 64 (52.1%) had a retained expression (score 2), 43 (34.4%) presented with decreased expression (score of 1), and 18 (14.4%) were Numb deficient (score of 0) (Figure 1). The associations between Numb expression and patient and tumour characteristics in the studied cohort are summarized in Table 1. The percentage of Ki67 > 14% in the retained Numb group was significantly lower than that in the decreased and deficient Numb groups (86.00% vs. 98.40%, *P* = .0171) (Table 1). No significant difference was noted in terms of age (χ2 test; *P* = .2796), tumour size (*χ* ^2^ test; *P* = .5911), lymph node status (*χ* ^2^ test; *P* = .6091) and histological type (Fisher's exact test; *P* = .5762) between the retained Numb and decreased and deficient Numb groups.

**Table 1:** Associations between Numb expression and patient and tumour characteristics

Factors	Triple‐negative tumours (n = 125)	Retained Numb n = 64 (51.2%)	Reduced and Deficient Numb n = 61 (48.8%)	*P* value
Age, years				
≤50	76	42	34	0.2796
>50	49	22	27	
Tumour size				
≤2 cm	54	26	28	0.5911
>2 cm	71	38	33	
Lymph node status				
Positive(n > 0)	41	27	23	0.6091
Negative(n = 0)	84	37	38	
Histological type				
DCIS	4	2	2	0.5762
IDC	112	57	55	
Medullary	2	0	2	
Other	6	4	2	
Missing	1	1	0	
Ki67				
≤14%	10 (8%)	9 (14%)	1 (1.6%)	0.0171*
>14%	115 (92%)	55 (86%)	60(98.4%)	

Note

The categorical variables were reported as numbers (n) and percentages of the total (%), and chi‐square test and Fisher's exact test were used to test the difference. The data analysis for this study was generated using SAS 9.4 software (Copyright^©^ 2016 SAS Institute Inc Cary). Significant difference was determined at the α level of 0.05. All P values were calculated using chi‐square test, except when calculating correlation between histological type, Ki67 and Numb expression when Fisher's exact was used. There is no significant difference in age, tumour size, lymph node status, histological type between the retained Numb group and reduced and deficient Numb group. The per cent of Ki67 > 14% in retained Numb group was significantly lower than that in the reduced and deficient Numb group (86.00% vs. 98.40%, *P* = .0171).

Abbreviations: DCIS, ductal carcinoma in situ; IDC, invasive ductal carcinoma.

* The *P* value is significant.

### 3.2 The localization and expression of Numb, HDM2 and p53 in MCF‐10A and MDA‐MB‐231 cell lines

**Figure 2:**
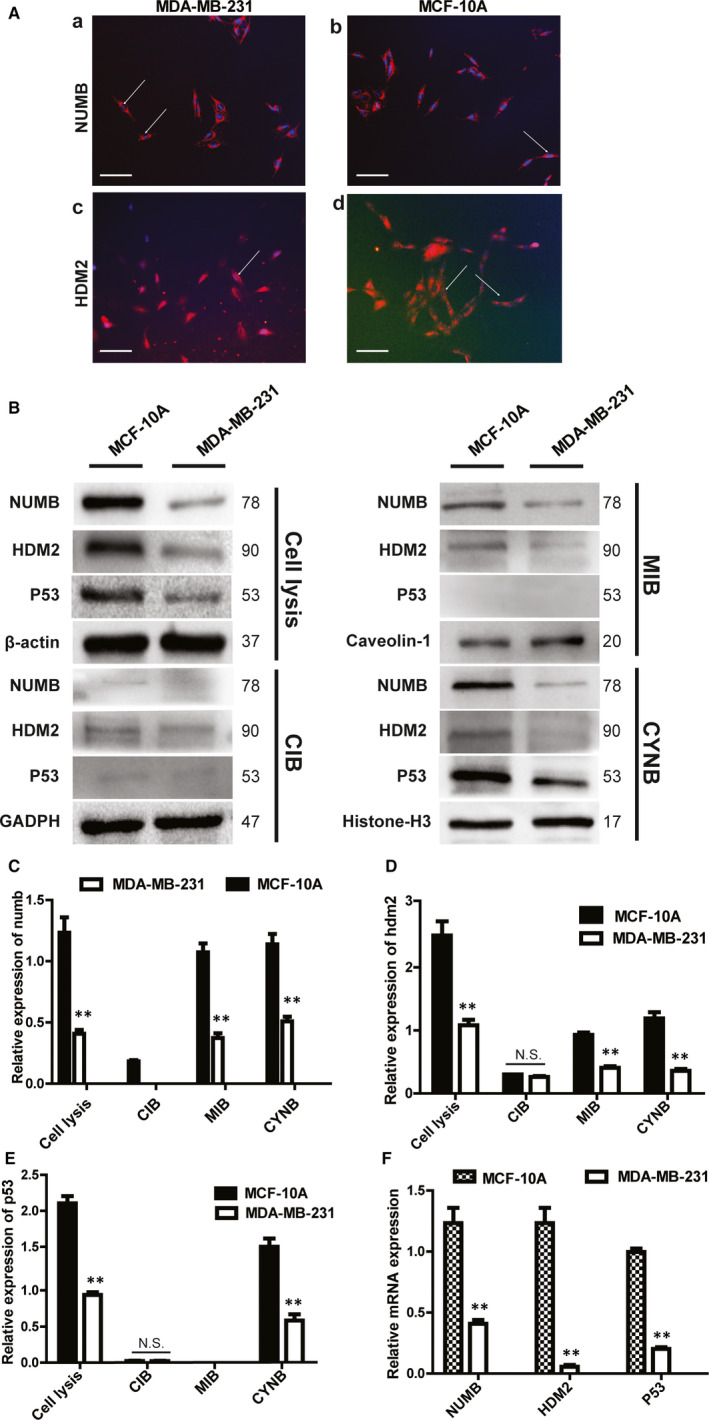
The localization and expression of Numb, HDM2 and p53 in MCF‐10A and MDA‐MB‐231 cell lines Cell lysis, whole‐cell protein; MIB, membrane fraction; CIB, cytoplasmic fraction; CYNB, nuclear fraction. A, Immunofluorescence staining of NUMB and HDM2. (a) The localization of NUMB in MDA‐MB‐231 (X400); (b) the localization of NUMB in MCF‐10A (X400); (c) the localization of HDM2 in MDA‐MB‐231 (X400); (d) the localization of HDM2 in MCF‐10A(X200) Scale bar represented 50 μm. B, The protein expression of NUMB, HDM2 and p53 in different cell fractions of MDA‐MB‐231 and MCF‐10A was determined by Western blot. C, D, E Quantitative analysis of NUMB, HDM2 and p53 expression in different cell fractions of MDA‐MB‐231 and MCF‐10A (Mean ± SD, n = 3, **P < .01). F, Quantitative RT–PCR detection of *NUMB, HDM2* and *p53* levels in MDA‐MB‐231 and MCF‐10A

Cellular immunofluorescence experiment was performed to locate the two proteins of Numb and HDM2 in the MCF‐10A and MDA‐MB‐231 cells. The results demonstrated that Numb was primarily distributed in the membrane fraction of both cell lines, and a weak fluorescence was detected in the nucleus (Figure 2A). HDM2 was distributed in the cell membrane, cytoplasm and nucleus of the MCF‐10A and MDA‐MB‐231 cells (Figure 2A). The mRNA expression of NUMB, P53 and HDM2 were higher in the MCF‐10A cells than in the MDA‐MB‐231 cells, and this trend is similar to the protein levels. (Figure 2B and F). We further isolated and extracted the cell fractions. Western blot experiments demonstrated that Numb and HDM2 were distributed in the cell membrane, cytoplasm and nucleus, whereas p53 was primarily distributed in the nucleus of the MCF‐10A cells (Figure 2B). The expression of Numb, HDM2 and p53 in the nuclear fraction was higher in the MCF‐10A cells than in the MDA‐MB‐231 cells (Figure 2B). In the membrane fraction, the protein level of Numb and HDM2 was higher in the MCF‐10A cells than in the MDA‐MB‐231 cells. However, p53 was not detected (Figure 2B). In the cytoplasmic fraction, the expression of Numb was significantly higher in the MCF‐10A cells than in the MDA‐MB‐231 cells. However, no difference was noted in the HDM2 and p53 protein levels (Figure 2B).

Importantly, the Numb levels were remarkably higher in all the cell fractions of the MCF‐10A cells than in the MDA‐MB‐231 cells (Figure 2C). The HDM2 levels of the cytoplasmic fraction were not different between these two cell lines. However, its expression was higher in the cell membrane and nucleus of the MCF‐10A cells than in those of the MDA‐MB‐231 cells (Figure 2D). The expression of p53 in the nuclear fraction was remarkably higher in the MCF‐10A cells than in the MDA‐MB‐231 cells. In the cytoplasmic fraction, p53 expression was extremely low, and no significant difference was noted between the MDA‐MB‐231 and MCF‐10A cells. In addition, p53 was not expressed in the cell membrane (Figure 2E).

### 3.3 Numb, HDM2 and p53 levels in the different cell fractions of the NUMB–EGFP‐transfected MDA‐MB‐231 cells

**Figure 3:**
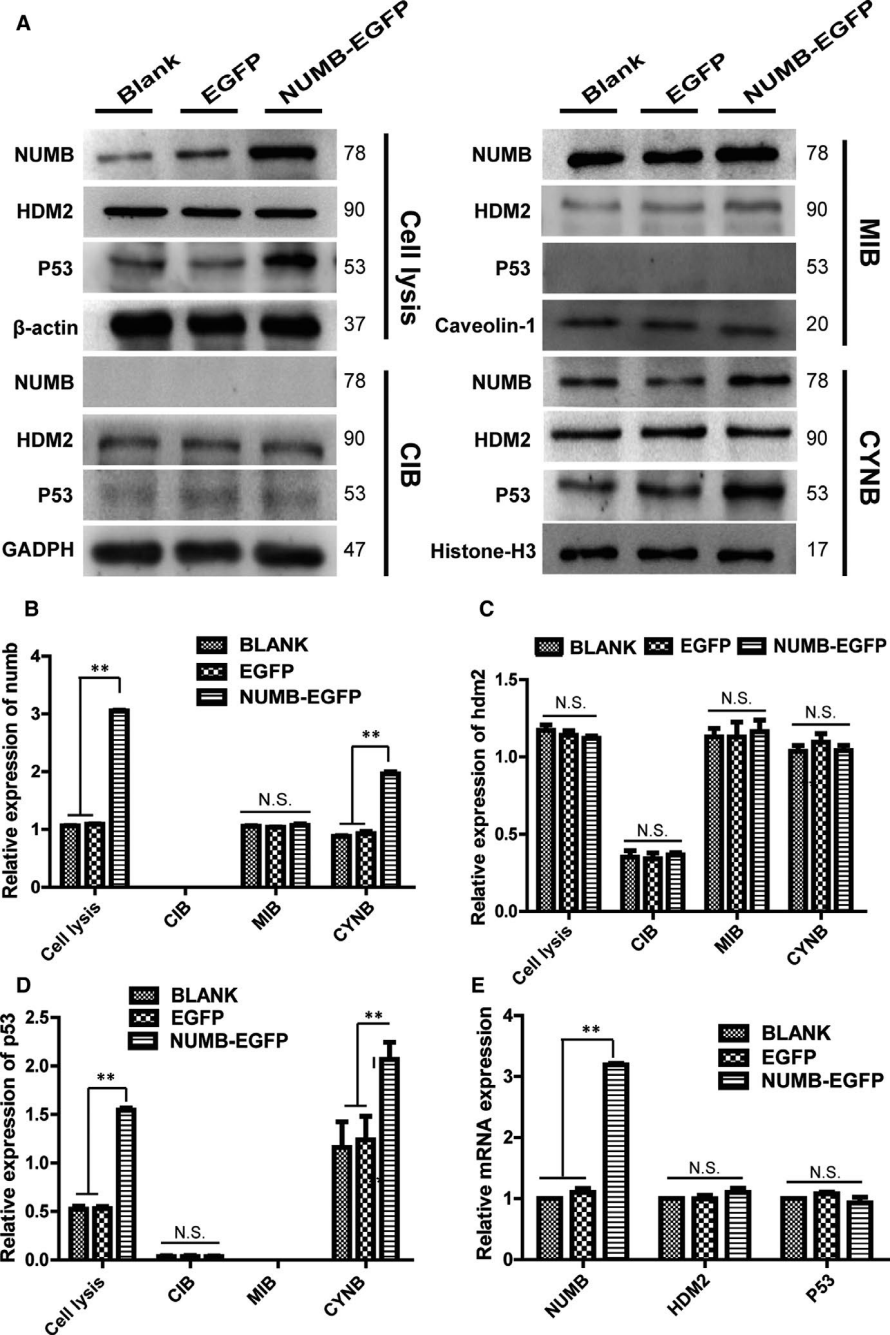
Numb, HDM2 and p53 levels in the different cell fractions of the NUMB–EGFP‐transfected MDA‐MB‐231 cells. A, The protein expression of Numb, HDM2 and p53 in the membrane, cytoplasmic and nuclear fractions of NUMB‐EGFP‐transfected MDA‐MB‐231 cells was determined by Western blot. B, C, D Quantitative analysis of Numb, HDM2 and p53 proteins in different cell fractions of NUMB‐EGFP‐transfected cells. (Mean ± SD, n = 3, **P < .01, vs blank group or EGFP group). E Quantitative RT–PCR detection of *NUMB, HDM2* and *p53* levels in NUMB‐EGFP‐transfected MDA‐MB‐231 cells

The above‐mentioned experimental data suggested that Numb was highly expressed in MCF‐10A and relatively low in the basal‐like cell line MDA‐MB‐231. Therefore, NUMB was re‐expressed in the MDA‐MB‐231 cells by transfecting with NUMB–EGFP plasmid. Furthermore, after separating different cell fractions for Western blot experiments, the expression of Numb and p53 significantly increased in the nuclear fraction of the NUMB–EGFP‐transfected MDA‐MB‐231 cells, whereas no changes were observed in HDM2 (Figure 3A). However, in the fraction of the membrane and cytoplasm, Numb, HDM2 and p53 have not been affected (Figure 3A). Meanwhile, no significant change was observed in HDM2 and P53 in the mRNA level after the re‐expression of NUMB in MDA‐MB‐231 (Figure 3E). Importantly, the expression of Numb in the membrane fraction did not significantly change, and in the cytoplasmic fraction, no expression was observed. Moreover, a significant increase was observed in the nuclear fraction after the re‐expression of NUMB in the MDA‐MB‐231 cells (Figure 3B). The HDM2 levels did not change in any of the fractions of the NUMB–EGFP‐transfected MDA‐MB‐231 cells (Figure 3C). The p53 levels in the nuclear fraction significantly increased, and no expression was observed in the membrane and cytoplasmic fractions of the NUMB–EGFP‐transfected MDA‐MB‐231 cells (Figure 3D).

### 3.4 Effects of NUMB knock‐down on Numb, HDM2 and p53 expression in the different cell fractions of MCF‐10A cells

**Figure 4:**
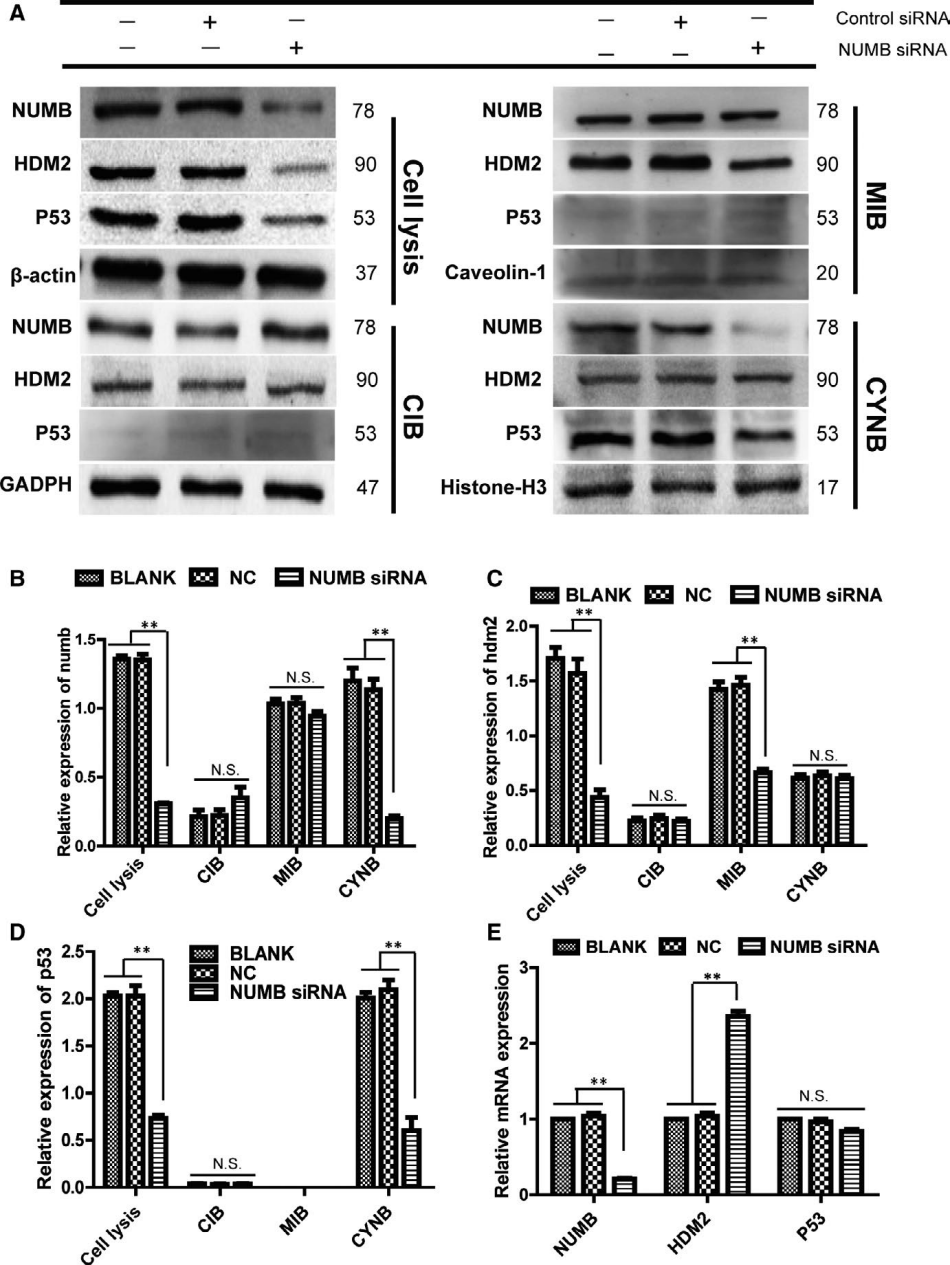
Effects of NUMB knockdown on Numb, HDM2 and p53 expression in the different cell fractions of MCF‐10A cells. A, The protein expression of Numb, HDM2 and p53 in the membrane, cytoplasmic and nuclear fractions of Numb knockdown MCF‐10A cells was determined by Western blot. B,C,D Quantitative analysis of Numb,HDM2 and P53 proteins in different cell fractions of Numb knockdown cells（Mean ± SD, n = 3 **P < .01, vs blank group or NC group）. E, The mRNA expression of *NUMB,HDM2* and *p53* in Numb knockdown cells was determined by q‐PCR

Transfection with NUMB siRNA decreased Numb expression in the MCF‐10A cell line, and the protein level of HDM2 and p53 decreased accordingly (Figure 4A). q‐PCR indicated that the mRNA level of HDM2 was significantly increased, whereas p53 did not change significantly (Figure 4E). Furthermore, after separating the cell fractions for Western blot experiments, the protein level of Numb and p53 both decreased in the nuclear fraction, whereas no changes were noted in HDM2 (Figure 4A). In the membrane fraction of MCF‐10A, Numb expression did not change significantly, whereas the HDM2 decreased remarkably. Moreover, p53 was not detected (Figure 4A). The Numb, HDM2 and p53 levels were not significantly different in the cytoplasmic fraction (Figure 4A). Importantly, the Numb levels were significantly decreased in the nuclear fraction after tampering with NUMB in the MCF‐10A cell line, and it did not significantly change in the membrane and cytoplasmic fractions (Figure 4B). The expression of HDM2 significantly decreased in the membrane fraction of MCF‐10A, and that in the cytoplasmic and nuclear fractions did not change (Figure 4C). The p53 levels in the nucleus of the MCF‐10A cells significantly increased and that in the cytoplasm remained unchanged. No expression was noted in the cell membrane (Figure 4D).

## 4 DISCUSSION

A higher percentage of tumours from the triple‐negative (SR‐/HER2‐) sub‐group displayed decreased or deficient Numb expression compared with those in the other sub‐groups (44% [11/25] of the basal‐like triple‐negative cell line).^18^ We performed immunohistochemical staining on the paraffin sections from 125 patients with TNBC to detect Numb expression.^19^, ^20^ The results showed that 64 (51.2%) patients had retained Numb expression and 61 (48.8%) presented with decreased or deficient Numb expression. This indicated that decreased or deficient Numb expression is common in TNBC. Furthermore, we analysed the associations between Numb expression and patient and tumour characteristics. The percentage of Ki67 >14% was significantly lower in the retained Numb group than in the decreased or deficient Numb groups (86.00% vs. 98.40%, *P* = .0171). Thus, an inverse correlation may be observed between Numb expression and indicator of tumour proliferation. Moreover, the loss of Numb expression was associated with poor prognosis; thus, its clinical relevance was further questioned.

Numb inhibits the degradation of p53 by HDM2 in the MCF‐10A cells, thereby maintaining the activity and stability of p53. However, whether these three proteins interact and their specific mechanisms of action remain unknown.^17^ Our results demonstrated that Numb and HDM2 were distributed in the membrane as well as in the cytoplasmic and nuclear fractions of the MCF‐10A cells, whereas p53 was primarily distributed in the nucleus. Numb was not detected in the cytoplasm of the MDA‐MB‐231 cells. We compared the transcription levels and protein expressions of NUMB, HDM2 and p53 in these two cell lines. The Numb and HDM2 levels were significantly higher in all the cell fractions of the MCF‐10A cells than in those of the MDA‐MB‐231 cells. In the nuclear fraction, the p53 level was higher in the MCF‐10A cells than in the MDA‐MB‐231 cells. Thus, we re‐expressed NUMB in the MDA‐MB‐231 cells with the NUMB–EGFP plasmid and then detected the Numb, HDM2 and p53 levels in each cell fraction. However, no significant change was noted in the cytoplasmic and membrane fractions of the three proteins after the re‐expression of NUMB. However, in the nuclear fraction, Numb and p53 levels were significantly increased, whereas no significant change was noted in the HDM2 levels. q‐PCR showed that the transcriptional levels of HDM2 and p53 did not change after the re‐expression of NUMB. The above‐mentioned results showed the re‐expression of NUMB in the MDA‐MB‐231 cells, and that Numb may migrate from the cell cytoplasm into the nucleus to prevent the ubiquitination and degradation of p53 by HDM2. Moreover, this finding is also consistent with previous studies showing the NUMB‐mediated regulation of p53 at the post‐transcriptional level in the MCF‐10A cells.^17^ After knocking down NUMB in the MCF‐10A cells, the protein levels of Numb, HDM2 and p53 decreased significantly. We then obtained cell fractions for further analysis, and results showed that the Numb levels significantly decreased in the nuclear fraction. However, it did not significantly change in the membrane and cytoplasmic fractions. In addition, the p53 levels significantly decreased in the nuclear fraction of the NUMB knock‐down cells. Furthermore, regardless if Numb is re‐expressed in the MDA‐MB‐231 cells or knocked down in the MCF‐10A cells, it regulates p53 levels in the nucleus where the protein levels of Numb is positively correlated with p53 protein levels. This finding is also consistent with previous studies showing that the decrease in p53 levels was caused by the loss of Numb by HDM2.^17^

Several questions need answers. We found that the level of HDM2 significantly decreased in the membrane fraction after knocking down NUMB in the MCF‐10A cells. However, mRNA levels were significantly increased. We assume that a significant increase in HDM2 levels at the transcription level may be due to feedback regulation caused by the decrease in HDM2 levels in the cell membrane of the NUMB knock‐down MCF‐10A cells. Therefore, we believe that the decrease in HDM2 is caused by the regulation of post‐transcriptional levels in NUMB knock‐down cells. However, the exact mechanism underlying the decrease in HDM2 levels in the cell membrane and the correlation between decreased HDM2 and decreased Numb levels in the nucleus of NUMB knock‐down cells are not fully elucidated.

## CONFLICT OF INTEREST

The authors declare no conflicts of interest.

## AUTHOR CONTRIBUTIONS

JX and XQ performed experimental work and analysed data. YC, YQL and JX performed data analysis and wrote the manuscript. Youde.C planned and supervised the project, provided samples and supervised the histopathological analysis.

## Data Availability

All data generated or analysed during this study are included in this published article and its additional information files.
